# Agreeing the content of a patient‐reported outcome measure for primary care: a Delphi consensus study

**DOI:** 10.1111/hex.12462

**Published:** 2016-04-28

**Authors:** Mairead Murphy, Sandra Hollinghurst, Chris Salisbury

**Affiliations:** ^1^ Centre for Academic Primary CareSchool for Social and Community MedicineUniversity of BristolBristolUK

**Keywords:** Delphi, consensus study, primary care, PROM, questionnaire

## Abstract

**Background:**

As the first contact for any health‐related need, primary care clinicians often address multiple patient problems, with a range of possible outcomes. There is currently no patient‐reported outcome measure (PROM) which covers this range of outcomes. Therefore, many research studies into primary care services use PROMs that do not capture the full impact of these services.

**Objective:**

The study aim was to identify outcomes sought by primary care patients which clinicians can influence, thus providing the basis for a new primary care PROM.

**Methods:**

We used a Delphi process starting with an outcomes list inductively derived in a prior qualitative study. Thirty‐five experts were recruited into patient, clinician and academic panels. Participants rated each outcome on whether it was (i) relevant to health, (ii) influenced by primary care and (iii) detectable by patients. In each round, outcomes which passed/failed preset levels of agreement were accepted/rejected. Remaining outcomes continued to the next round.

**Results:**

The process resulted in a set of outcomes occupying the domains of health status, health empowerment (internal and external), and health perceptions. Twenty‐six of 36 outcomes were accepted for inclusion in a PROM. Primary care having insufficient influence was the main reason for exclusion.

**Conclusions:**

To our knowledge, this is the first time PROM outcomes have been agreed through criteria which explicitly exclude outcomes less relevant to health, uninfluenced by primary care or undetected by patients. The PROM in development covers a unique set of outcomes and offers an opportunity for enhanced research into primary care.

## Background

### Introduction

Primary care has been evolving in recent years to meet changing population and service needs as well as public expectations. In the USA, the 2010 Patient Protection and Affordable Care Act initiated a transformation of the health‐care system which emphasizes preventative, community and primary care.^1^ In the UK, new models of care have been introduced to improve access^2^, ^3^, ^4^ and interventions have been piloted to improve care for people with multiple long‐term conditions^5^, ^6^, ^7^, ^8^ as health services globally are challenged by increasing multimorbidity.^9^, ^10^

Assessing the effectiveness of such interventions from a patient perspective requires use of a patient‐reported outcome measure (PROM), i.e. a questionnaire that captures outcomes as experienced by the patient completing them. An ‘outcome’ has been defined as change in a patient's health status, knowledge or behaviour which is attributable to preceding health care,^11^ and PROMs provide an invaluable source of evidence for this change from the patient's point of view.^12^

Most PROMs are disease‐specific, that is, tailored to the symptoms and functional impacts of a particular condition.^13^ These cannot measure the effectiveness of interventions in primary care where patients could have a wide range of conditions. Primary care services are first‐contact, comprehensive and coordinating^14^ and thus require a *generic* PROM, which can be administered across a population, regardless of condition. Such a PROM should be based on outcomes that are relevant to patients, and that primary care clinicians can influence.^15^

A key problem with most generic PROMs used for primary care is responsiveness to change.^16^ The EQ‐5D^17^ and the SF‐36^18^ often show no change following interventions in primary care.^19^, ^20^, ^21^ This is because primary care patients frequently present with problems unrelated to symptoms or function,^22^ and many have multiple long‐term conditions^9^, ^10^, ^23^ so improving their function may not be possible. Outcomes such as a sense of control and the ability to self‐care may be more relevant for such patients.^24^

This issue was recognized 20 years ago, when the Measure Yourself Medical Outcome Profile (MYMOP) was designed. An individualized PROM which allows patients to define the symptoms and activities to be measured, MYMOP shows change when other PROMs do not.^21^, ^25^ However, its individualized nature means it has to be administered at interview with a clinician, making it pragmatically unfeasible for use in many research studies, and its focus on symptoms and function is narrow. The six‐item Patient Enablement Measure (PEI), developed shortly after MYMOP, encompasses broader outcomes, including coping, understanding and confidence in health.^26^ It has been well validated for primary care and is short and acceptable to patients and practitioners.^27^ However, as well as omitting symptoms and function altogether, it requires patients to assess change from a previous point in time, and attribute this to an intervention, a task which many questionnaire respondents find difficult.^28^

Some generic PROMs have attempted to deal with the problem of responsiveness to change by defining domains which, although they apply to people with a range of conditions, are particularly sensitive to certain interventions. For example, the adult social care outcomes toolkit (ASCOT) is a short PROM which measures health‐related quality of life in older people, with the stated aim of being sensitive to outcomes of social care.^29^, ^30^ We believe there is a need for a new PROM for primary care, which similarly focusses on the outcomes patients want from primary care, and which clinicians can influence.^31^

### Previous qualitative study

Establishing a clear construct through consultation with stakeholders is a necessary first step in the development of PROMs.^32^, ^33^ We previously carried out a qualitative interview study with primary care patients and clinicians to establish the outcomes which both groups sought to achieve.^34^ In that study, we used a broad definition of *Primary Care Outcome* as any effect of primary care on a patient's health or ability to impact health. We considered health in its widest sense, using the World Health Organisation (WHO) definition of health as ‘a state of complete physical, mental and social well‐being and not merely the absence of disease or infirmity’.^35^ We focussed our analysis on patient *outcomes from care* (as opposed to patient desired *experiences of care*) and identified and categorized 31 interrelated outcomes into 10 groups occupying four domains:

1. Health Status: This involves both 1) symptoms and medication side‐effects and 2) the impact of symptoms on patients’ lives.
2. Health Empowerment (Internal): These are the internal resources which enable patients to improve their health. This involves 3) patients understanding their illnesses/problems, 4) agreeing and adhering to a patient‐clinician shared plan, 5) being able to self‐care and stay healthy.
3. Health Empowerment (External): These are the external resources which enable patients to improve their health. This involves 6) patients having confidence in seeking health care and 7) access to suitable health‐related support.
4. Health Perceptions: This involves 8) patients’ satisfaction with their health, 9) health concerns and 10) confidence in their health for the future.

Health Status is the main reason for providing health care, but one which primary care cannot always influence. Its continuous, coordinating and comprehensive nature puts primary care in a unique position to additionally impact domains 2–4 over time. Although these are not traditionally viewed as outcomes, the previous study results suggested that they can be enduring impacts of primary care that have a direct influence on patients overall health status and are qualitatively different from measures of patient experience.^34^

### The Delphi consensus technique

The Delphi technique is a widely used method for achieving consensus. It uses a series of questionnaires to collect information from participants in a number of iterations, or ‘rounds’. The starting point is an open questionnaire, or, in the case of a modified Delphi, a pre‐derived list of questions. Following each round, each participant receives an individualized report, which compares their responses to the group response. In subsequent rounds they can then reassess their responses in the light of this information. This process allows a controlled debate to take place, and consensus to build without necessitating a group interaction. This removes the time and resource required for this, and the bias resulting from dominant individuals.^36^, ^37^

This study used the Delphi technique to agree a list of outcomes suitable for inclusion in a PROM for primary care.

## Methods

We used a modified Delphi process, starting with the outcomes from the previous qualitative study in lieu of an open questionnaire. We chose the Delphi method, because at least two existing PROMS for primary care (PEI and MYMOP) have employed very different domains, and Delphi is particularly useful in areas on which there is no existing scientific agreement or where contention might be expected.^38^ We recruited participants in three groups: patients (the ultimate owners of health outcomes), clinicians (who deliver health outcomes) and academics (who study health outcomes). Purposive sampling was used to ensure a breadth of opinion. The academic panel was comprised of clinical and non‐clinical academics who were geographically spread across the UK, and had a reputation and publication record in the area of the development of primary care outcomes or PROMs. Patients and clinicians were recruited from 11 health centres in Bristol, and included participants from the previous qualitative study (continuing members) as well as new members. Continuing members were recruited directly by the researcher. New patient participants were recruited either by the practice manager through their membership of the practice patient participation group (PPG), or by a practice nurse in the case of non‐PPG members. Patients recruited were invited based on their interest in improving quality of primary care. Because the study required participants to think generically and respond to a relatively complex survey, the patient panel was a relatively well‐educated sample. New clinicians were recruited by the practice manager from seven health centres with a range of deprivation scores, including three below the lower quartile of deprivation to ensure a more deprived patient demographic was well represented.

We held three rounds: two questionnaires administered online and a face‐to‐face meeting. Questionnaire participants were asked to give each outcome ratings based on three respective criteria:

1. Relevance: Does the outcome relate directly to a patient's *health status*, or their *ability to impact their health*, according to the WHO definition of health?
2. Influenceability: Can the outcome be influenced by primary care?
3. Detectability: Can the outcome be directly detected by patients?

These assessment criteria were designed for this study and informed by criteria for quality indicators published by the RAND corporation^39^ the Institute of Medicine^40^ and Campbell and Roland, for UK general practice.^41^

### Round one methods

#### Data collection

The questionnaires showed the three criteria in columns, and the outcomes in rows. Participants were invited to respond ‘Yes’, ‘Maybe’ or ‘No’ to each criterion for each outcome. They were also invited to suggest additional outcomes if they felt there were any missing from the list.

#### Data analysis

**Figure 1:**
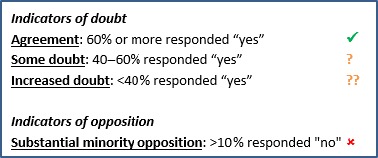
Outcome–criterion indicators.

Survey responses were analysed based on the presence of (i) doubt and (ii) opposition. Doubt was analysed by looking at the ‘yes’ responses. More than 60% of respondents responding ‘yes’ to a particular criterion was categorized as ‘little doubt’. Increasing levels of doubt were characterized by fewer respondents indicating ‘yes’. Opposition was characterized by a substantial minority of respondents indicating ‘no’ to a particular criterion. To ensure panels with more members did not exert undue influence, we calculated the unweighted mean of the panels. The indicators developed for each outcome and criterion are shown in Fig. 1.

The aim was to *accept* those outcomes where there was broad agreement and no opposition, *reject* those with both doubt and opposition and *carry forward* those with more doubt, or views polarized between agreement and opposition. We therefore accepted outcomes in round one if they had no (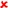) indicators and maximum of one (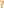) indicator (i.e. 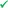 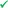 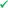 or 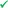 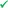 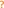 in any order). We rejected outcomes with an opposition indicator (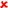) and a doubt indicator (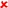 or 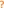 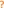) for any of the three criteria. All other outcomes continued to round two, along with the new outcomes suggested.

**Figure 2:**
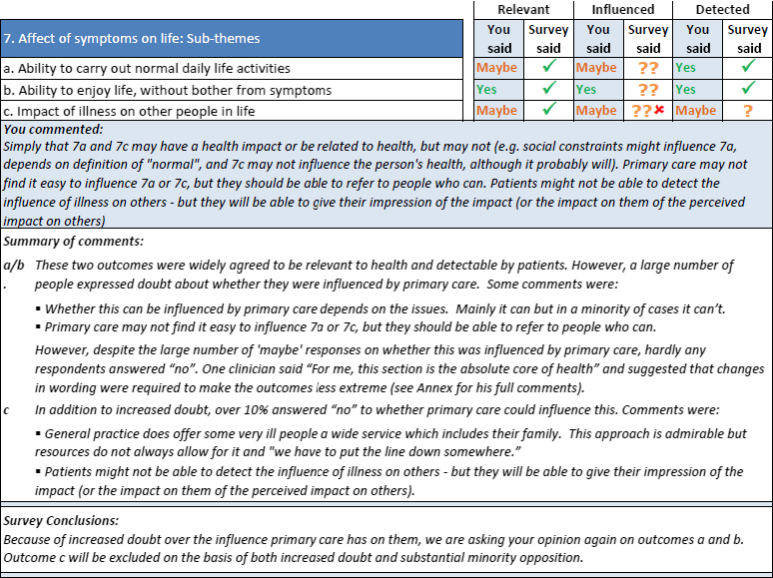
Example section from personalized Delphi report.

Each participant received an individualized report which contained, by outcome and criterion, their response compared to the overall response. A section of such a report is shown in Fig. 2.

### Round two methods

#### Data collection

The uncertain outcomes from round 1 were included in a new questionnaire with the new outcomes suggested added. Participants to this were in a similar way to round 1. The questionnaire structure was identical to previous round except that, in response to the finding that the 3‐point scale would benefit from more response options (see results), the range of responses was increased from three to five: ‘Yes’, ‘Mostly’, ‘Sometimes’, ‘Rarely’ and ‘No’.

#### Data analysis

The questionnaires were aggregated. ‘Yes/Mostly’ responses were considered to indicate agreement (the equivalent of ‘Yes’ in round 1), ‘Sometimes’ to indicate ‘doubt’ and ‘rarely/no’ responses ‘opposition’ (the equivalent to ‘no’ in round 1). Because the introduction of five response categories meant outcomes were less likely to be categorized as doubtful, we used a stricter acceptance condition in round two than round one, retaining outcomes only where all three criteria showed agreement. Apart from this, the criteria remained the same. Similar individualized reports were created for round two as for round one.

We used the McNemar test to evaluate opinion shift between the rounds. A modification of the paired t‐test, this determines whether the percentage of respondents who become more positive on a given item differs significantly from the percentage who become more negative.^37^

### Round three methods

Outcomes which were uncertain at the end of round two were reviewed in a face‐to‐face meeting to which all round 2 participants were invited. For each uncertain outcome, attendees were provided with the round 1 and 2 details, and with a sample question item which exemplified this outcome. There were then asked to respond ‘yes’ or ‘no’ to a ‘consensus question’. The consensus question asked was one of the following 3, depending on the criterion with most doubt at the end of round 2:

#### Consensus questions

1. Relevance: Do you think an improvement in this question item is tantamount to an improvement of a patient's health status, or their ability to impact their health status for most patients?
2. Influencability: Imagine a person scores the mid‐point to this question. Do you think receiving good primary care for one year would make her more likely to score higher next time than she would do if she received poorer quality primary care for one year?
3. Detectability: Do you think this question item would be meaningful to patients and they would be able to answer it in a way that fairly reflected the care they had been given?

If any outcome had a majority of ‘No’ responses after this process, it was excluded; otherwise, it was accepted.

## Results

### Round one results

#### Overview

There were 35 participants in round 1: nine academics, 12 clinicians and 14 patients. A description of the participants is shown in Table 1. This table shows a broad representation of participants, although there were more women than men, and more GPs than nurses.

**Table 1:** Characteristics of academic, clinician and patient Delphi participants

	Number of participants		
	Academic	Clinician	Patient
Gender			
Male	3	5	4
Female	6	7	10
Health Centre (IMD decile)			
HC 1 (8)	–	1	0
HC 2 (6)	–	3	0
HC 3 (5)	–	1	1
HC 4 (4)	–	2	2
HC 5 (1)	–	2	0
HC 6 (1)	–	1	0
HC 7 (1)	–	2	0
HC 8 (2)	–	0	1
HC 9 (2)	–	0	2
HC 10 (9)	–	0	4
HC 11 (4)	–	0	4
Academic or Clinical Role			
Professor/Director	4	–	–
Other career level	5	–	–
GP	–	10	–
Practice Nurse	–	2	–
Academic background			
Medical	3	–	–
Other Clinical	1	–	–
Non‐clinical	5	–	–
Clinician years since qualification			
More than 20	–	6	–
10–20	–	5	–
<10	–	1	–
Patient long‐term health conditions			
>1 LTC	–	–	5
1 LTC	–	–	4
None	–	–	3
Not disclosed	–	–	2

Participants were generally favourable towards the outcomes, with more than 60% ‘yes’ responses. The remainder were nearly all ‘maybe’, with <3.5% ‘no’ responses in the entire questionnaire. More doubt was expressed about the extent to which the outcomes could be *influenced* by primary care than the other two criteria.

There was little difference between the three panels. Although clinicians gave slightly less positive responses than either patients or academics, this was not statistically significant, apart from in the *detected* criterion, where a chi‐squared test showed academics giving relatively more ‘yes’ responses than clinicians and patients. At an individual outcome level significant differences between panels were observed on only three of 93 question/response pairs which had a chi‐squared *P*‐value below 0.05: for

1. pain (*detected* by patients)
2. other signs and symptoms (*detected* by patients)
3. dealing with the root cause of illness (*influenced* by primary care)

For all of these questions, clinicians, and sometimes patients, showed a greater tendency to respond negatively than academics.

#### Outcomes accepted

Sixteen outcomes were accepted for inclusion in a pilot PROM in round 1. These included outcomes on patient concern, and many of the internal empowerment outcomes in groups 3–5, such as patients’ understanding of their illnesses, and their ability to self‐care, stay healthy and manage symptoms. In support of outcomes on patient understanding, one participant explained:

Patient's insight depends on … healthcare professional's ability to translate information into patient speak and checking patients’ understanding. (Patient 3)

By referring to the role of health professionals, this participant was explaining his response that this outcome could be *influenced* by primary care. Some participants, while also responding positively, added a caveat to note that there would be exceptions to this:

Some patients do not have either the ability or desire to understand. (Patient 13)

This patient felt that there would be certain patients for whom understanding was not important, and therefore less *relevant* to their health.

The group 1 outcomes on physical and emotional symptoms were also accepted. Pain/discomfort was given an indicator of agreement on all three criteria. Anxiety, depression and stress had an indicator of doubt on the *influence* of primary care. A participant commented:

As some of root causes of anxiety/depression/stress and other symptoms might not be obviously health‐related (e.g. finances, family trouble), primary care may be less able to help. (Academic 5)

However, there was only doubt on this one criterion, and little opposition, so these first three outcomes were all accepted in round one.

#### Other outcomes

Three outcomes were rejected in round 1. More doubt was expressed about outcome 4c (patients take responsibility for their own health) than any other, because of the perceived limited *influence* of primary care. Only 25% of participants responded ‘yes’ on whether it could be *influenced* by primary care and 16% responded ‘no’. These views were consistent across all three panels.

Similarly, only 16% of participants responded ‘yes’ on whether outcome 3c, the impact of illness on other people in a patient's life, could be *influenced* by primary care, and over 10% answered ‘no’. One participant commented:

General practice does offer some very ill people a wide service which includes their family. This approach is admirable but resources do not always allow for it. (Academic 7)

Twelve outcomes remained uncertain in round 1, including the effect of symptoms on life. There were five new outcomes suggested, extracted from a list of 31. The 26 suggested but not included in round 2 were measures of process, or best measured through health‐care information systems, including continuity of care, access to language support and waiting times.

### Round two results

Thirty (86%) participants responded to round two: eight academics, 10 clinicians and 12 patients. As with round one, responses were generally favourable towards the outcomes, with more than 60% being ‘yes’ or ‘mostly’, and with the most doubt expressed about the extent to which the outcomes could be *influenced* by primacy care.

Because yes/mostly and rarely/no were aggregated in round 2, one‐point movements from ‘yes’ and ‘no’ in round 1 to ‘mostly’ and ‘rarely’ in round 2 were not counted as opinion change, but one‐point shifts from ‘maybe’ in round 1 were. All changes of two points or more were counted as opinion shift. Opinion shifted in a positive direction between rounds one and two. Three McNemar tests were carried out on three 2 × 2 contingency tables with doubtful and negative responses grouped. This showed that the positive shift was significant (*P* ≤ 0.05 for all 3 criteria). This analysis grouped all 12 outcomes together. Analysis of individual outcomes showed significant shifts for only a few outcomes.

#### Outcomes accepted

Five of the 17 outcomes were accepted in round 2, in part because participants became more positive about the *relevance* of some outcomes. Clinician 1 explained this change from his point of view.

I have changed my view on some of these. The reason is that I am now including (as I suspect I was only partially before) a kind of indirect influence. (Clinician 1)

This clinician widened his internal definition of ‘health outcome’ in round two, and so responded more positively on the criterion of *relevance* for some of the empowerment outcomes.

#### Other outcomes

Four outcomes were rejected in this round: three of these being outcomes which were suggested in round 1. Eight outcomes were still uncertain, seven of which had indicators of doubt (<60% yes) on the *influencability* criterion. For example, for outcomes 2a and 2b (the effect of symptoms on patient's lives), participants focussed on the reduced impact of primary care:

[I am] casting some doubt over primary care's influence, as other services and factors in people's lives (such as social support) will influence these outcomes as well. (Academic 5)

It is notable that, despite a large number of ‘sometimes’ responses, no participants responded ‘rarely’ or ‘no’ on this criterion for these outcomes. Another clinician, who responded ‘yes’ to all criteria for 2a and 2b said:

I have changed my view very little here. In truth I was completely amazed at the lack of consensus here from others. What kind of medicine are they practising? (Clinician 1)

This clinician pointed out that influencing the effect of symptoms on patients’ lives may be a long‐term process, and as timescale was not incorporated in the Delphi questionnaire, participants may have found this criterion difficult to interpret.

### Round three results

**Figure 3:**
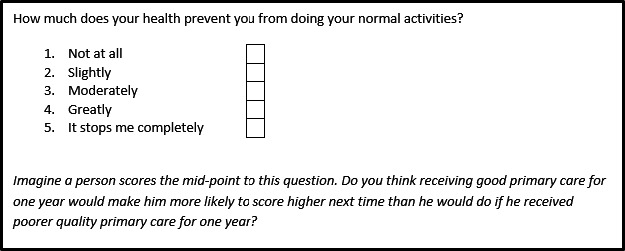
Round 3 sample item and consensus question.

The thirty participants from round 2 were invited to attend the final face‐to‐face meeting to discuss the eight uncertain outcomes. Six participants attended this meeting: 4 patients, 1 GP and 1 academic. The meeting was facilitated by the principal researcher and attended by a coresearcher. For each item, participants were given a handout with round 1 and 2 details, a sample item and a consensus question. For example, for outcome 7a, patients were shown the following the item and consensus question shown in Fig. 3.

Each participant gave their opinion in turn. These were then verbally summarized by the meeting facilitator and participants were invited to add to this summary or ask clarification questions. Each participant was then asked to respond ‘yes’ or ‘no’ to the consensus question. After discussion, all six participants responded ‘yes’ to the consensus question, and outcome 7a was accepted.

Four outcomes were accepted unanimously following this process, three excluded (with >50% ‘no’ responses), and one accepted by the majority, to be reviewed at PROM development stage. This was outcome 8b, about health damage due to poor medical care: some participants felt this should specify primary care.

Because of the low number of participants in this round, the results were reviewed carefully by the research team. Clear reasons were given for the 3 outcomes rejected, mostly relating to the limited influence of primary care. The five outcomes accepted will be taken forward into a PROM (with the outcomes accepted in the previous two rounds) and the items reviewed at questionnaire testing phase for patient comprehension.

### Overview of results

**Figure 4:**
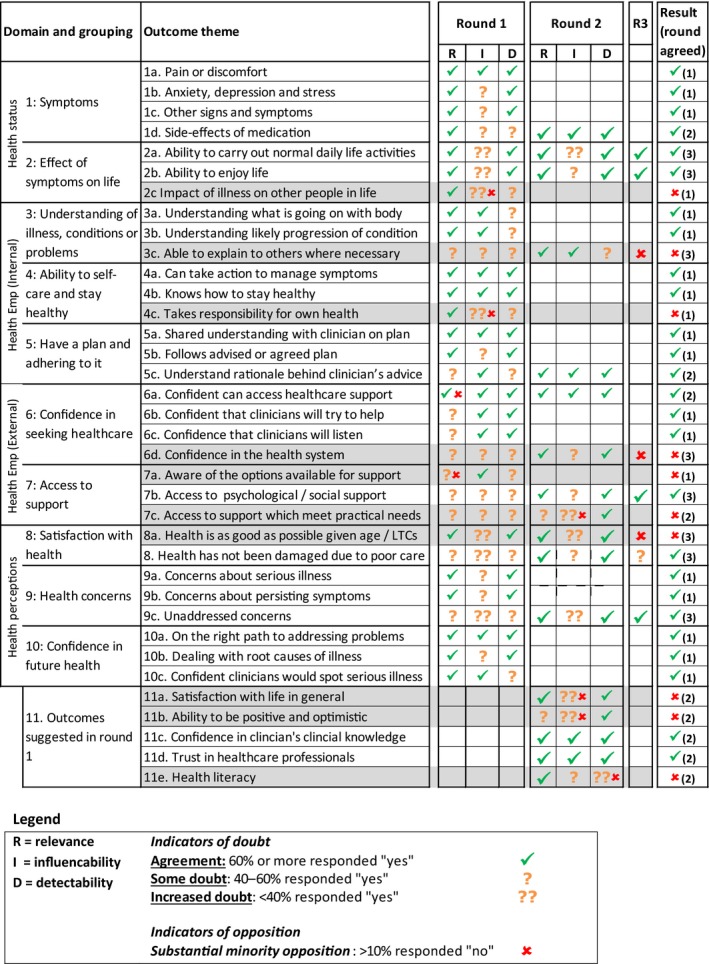
Results of the 3 Delphi rounds.

The list of outcomes considered in the Delphi exercise is shown in Fig. 4. The final column shows whether the outcome was excluded (cross) or taken forward (tick). Excluded outcomes are also shaded grey. All other outcomes were taken forward for inclusion in a PROM.

## Discussion

### Main findings

To our knowledge, this is the first time PROM outcomes have been agreed through criteria which explicitly exclude outcomes less relevant to health, uninfluenced by primary care, or undetected by patients. Of 36 outcomes, 26 (72%) were accepted for inclusion in the pilot PROM (24 from the original list of 31, and two that were suggested as part of the process). This is a relatively high proportion compared to some other Delphi studies, which may be due to the rigour of the prior qualitative phase, designed as it was to elicit primary care sensitive outcomes. It is comparable to a recent study which employed a similarly thematically derived list in round 1.^42^

We identified a large amount of commonality among the panels. This result differs from some other Delphi studies which found that patient and clinician views often differ.^43^, ^44^ This may be because we were investigating outcomes: a previous study which noted differences between patients and clinicians was investigating process indicators of quality, not outcomes.^44^ The core of the primary care consultation is a patient/clinician discussion with a view to achieving an outcome for the patient. Given this, it seems less surprising that the three panels should agree on what these outcomes are.

### Health status

The outcomes which reached consensus most quickly in this category were those on symptoms. The functional outcomes (ability to carry out normal activities/enjoy life) were not accepted until round three, and were subject to some debate. While on the one hand this seemed surprising (given that these outcomes are integral to health as we understand it), it reflects the reality that, despite the fact that improved health status is normally seen as the ultimate goal of health care, quality of care does not necessarily lead to this.^45^, ^46^ Indeed, the relative unresponsiveness of measures like the EQ‐5D and SF‐36 was one of the reasons for undertaking this study. Comments from clinicians suggested that their reluctance to accept these outcomes stemmed from concerns about measuring quality of care using domains not fully in their control. The use of the example item and the consensus question in round three helped to achieve consensus in this area.

This domain is also notable for the outcomes which are not included. The WHO definition of health includes social health and, because of this, health status measures based on this definition often contain items related to social health such the Duke Health Profile ‘I am happy with my family relationships’.^47^ Other health questionnaires focus on overall quality of life, such as the ICE‐CAP, which includes questions about love friendship and support, and enjoyment and pleasure.^48^

Such outcomes were not raised in the prior qualitative study. However, at the end of the first round, participants included the more general outcome ‘satisfaction with life in general’ and also ‘ability to be positive and optimistic’. Both were excluded in round two on the basis they were not sufficiently influenced by primary care. These results corroborate the findings of the initial qualitative work.

### Health empowerment (internal)

The outcomes of patient understanding, and ability to self‐care and stay healthy were accepted in round one. Participants noted that there would be exceptions where patients chose to limit their understanding. This was also noted in our prior qualitative study, and other research^49^ and will need to be taken account of when developing a PROM. The outcomes ‘ability to explain health problems to others’, and ‘patient takes responsibility for own health’ were excluded. A similar item to the latter *is* included in some other health empowerment measures. For example, the item ‘I am the person who is responsible for taking care of my health’ is the first item in the Patient Activation Measure.^50^ Although PAM overall has shown change following intervention,^51^, ^52^ the Delphi participants considered this particular outcome too difficult to influence through intervention in primary care.

### Health empowerment (external)

The external empowerment domain included outcomes relating to patients’ ability to access suitable primary health care clinicians and other health‐related supports. Such outcomes are not commonly included in PROMs, and some of these were questioned in round one with regard to their *relevance* – even given the definition of outcome as including patient's ability to improve their own health. In round two, participants were more positive about the *relevance* of most of these outcomes.

### Health perceptions

These included patient satisfaction with their health, health concerns, and confidence that they are on track for the future. A key outcome in this group rejected in round 3 was patient's perception of their own health, exemplified by the question: ‘All things considered, how would you rate your health for your age and situation?’ The majority of participants present thought primary care could not *influence* this beyond influencing outcomes of symptoms and function. Although all the other outcomes were accepted, exclusion of this outcome has changed the nature of the health perceptions construct, such that it now relates more to health concerns and confidence in a health‐care plan. Given that this outcome was rejected on the grounds of the *influence* of primary care, it is hoped that the remaining construct will be more responsive to change in primary care.

### Strengths and limitations

#### Strengths

The Delphi process has been used to establish quality indicators for primary care and attributes of primary care.^41^, ^53^ It is less frequently used to agree the content of PROMs. The three criteria we developed for this purpose were novel, acceptable to panels, and allowed for differentiation among the outcomes. The method was highly successful in simulating a conversation between experts. One issue with Delphi studies can be high dropout rates, and investigators have a key role in mitigating this.^36^ We maintained an 86% response rate between rounds one and two, despite the questionnaire having the same items and structure, which could have led to response fatigue. We believe that the high response rate between these two rounds resulted partly from our method of reporting indicators, and from the individualized reports, both of which helped to engage participants in the process. The lower response rate in round 3 is discussed in the limitations section.

The creator of the Delphi Method suggested three benefits of Delphi were removing the influence of dominant individuals, reducing noise and reducing the group pressure for conformity. These were particular relevant in the case of this study. In terms of noise reduction, much of the ‘communication’ in a discussion group often has to do with individual agendas, and is often irrelevant or biasing.^54^ On recruitment of the participants the researcher noted that many of them had a particular interest or agenda related to the overall topic of quality in primary care, but not directly related to outcomes. Many of these topics arose when participants raised additional outcomes in round one. Ensuring continuity of care, GP out‐of‐hours services and concerns about future funding of the NHS were some of the topics raised. The Delphi process was highly successful in filtering this ‘noise’, ensuring the conversation focussed on outcomes.

#### Limitations

Our questionnaire was relatively complex and long. We were open with participants about the complexity of the task and the time‐commitment required. This helped ensure a high follow‐up rate, but may have discouraged some participants who felt they did not have the time, or the necessary intellectual rigour to complete the questionnaire.

Some participants struggled with applying a generic response to something which would always be specific to a patient. This may have been exacerbated by the three‐point scale in the first round. Many Delphi studies use a scale with a relatively large number of response options.^41^, ^55^ We chose a three‐point scale partly to reduce the complexity of the task, and partly because of our analysis method chosen: which was a separate reporting of each response category in the form of indicators, an approach which has been used successfully in other studies.^55^ Although this approach was largely successful, some participants tended to select the middle option in round one ‘Maybe’ and commence their textual answer with ‘It depends’. The addition of five response options in the second round helped to resolve this.

Limitations of Delphi studies include the potential of investigators ‘moulding’ responses. Our reports could, in theory, have been designed to lead participants down a particular route. In practice, we endeavoured to remain objective when writing the qualitative reporting sections. We also made an Annex available with all unedited comments, although only one participant requested this Annex.

The final limitation relates to the number of participants in round three. Because of the necessity for a face‐to‐face meeting, these last eight outcomes were decided by a group of six people, including only one clinician. A different subset may have come up with different results. For example, the outcome ‘unaddressed concerns’ was accepted in round three. However, there were some clinicians who had been quite opposed to this outcome at the end of round two. Had they been present at the meeting, the decision on this outcome could have been different. The number of outcomes which was discussed at this final consensus meeting was relatively small and, given that they were still in doubt after 2 rounds, it was clear that there was no easy answer on inclusion or exclusion.

## Conclusions

The Delphi process was highly successful in engaging experts to test the results of the qualitative study. It employed a novel approach, using three criteria, and individualized indicator reports which kept participants engaged. The process has led to a set of outcomes which experts believe to be relevant to health, influenceable by primary care and detected by patients. It therefore provides a strong conceptual basis for a valid and responsive PROM for primary care. We plan to develop a PROM from this basis, and test its reliability and validity using established scale development procedures.^32^ The resultant PROM could then be used to assess the outcome of primary care interventions from a patient perspective.

## Funding source

This study was funded by the National Institute for Health Research School for Primary Care Research (NIHR SPCR). The NIHR SPCR is a partnership between the universities of Birmingham, Bristol, Cambridge, Keele, Manchester, Newcastle, Nottingham, Oxford and Southampton and University College London. The views expressed are those of the author(s) and not necessarily those of the NHS, the NIHR or the Department of Health.

## Conflict of interest

The authors have no competing interests.
